# Social Robots as Creativity Eliciting Agents

**DOI:** 10.3389/frobt.2021.673730

**Published:** 2021-09-13

**Authors:** Safinah Ali, Nisha Devasia, Hae Won Park, Cynthia Breazeal

**Affiliations:** MIT Media Lab, Massachusetts Institute of Technology, Cambridge, MA, United States

**Keywords:** creativity, social robots, emulation, scaffolding, creativity support tools, child-robot interaction, collaboration

## Abstract

Can robots help children be more creative? In this work, we posit social robots as *creativity support tools* for children in collaborative interactions. Children learn creative expressions and behaviors through social interactions with others during playful and collaborative tasks, and socially emulate their peers’ and teachers’ creativity. Social robots have a unique ability to engage in social and emotional interactions with children that can be leveraged to foster creative expression. We focus on two types of social interactions: *creativity demonstration*, where the robot exhibits creative behaviors, and *creativity scaffolding*, where the robot poses challenges, suggests ideas, provides positive reinforcement, and asks questions to scaffold children’s creativity. We situate our research in three playful and collaborative tasks - the Droodle Creativity game (that affords verbal creativity), the MagicDraw game (that affords figural creativity), and the WeDo construction task (that affords constructional creativity), that children play with Jibo, a social robot. To evaluate the efficacy of the robot’s social behaviors in enhancing creative behavior and expression in children, we ran three randomized controlled trials with 169 children in the 5–10 yr old age group. In the first two tasks, the robot exhibited creativity demonstration behaviors. We found that children who interacted with the robot exhibiting high verbal creativity in the Droodle game and high figural creativity in the MagicDraw game also exhibited significantly higher creativity than a control group of participants who interacted with a robot that did not express creativity (*p* < 0.05*). In the WeDo construction task, children who interacted with the robot that expressed creative scaffolding behaviors (asking reflective questions, generating ideas and challenges, and providing positive reinforcement) demonstrated higher creativity than participants in the control group by expressing a greater number of ideas, more original ideas, and more varied use of available materials (*p* < 0.05*). We found that both creativity demonstration and creativity scaffolding can be leveraged as social mechanisms for eliciting creativity in children using a social robot. From our findings, we suggest design guidelines for pedagogical tools and social agent interactions to better support children’s creativity.

## Introduction

Children’s creativity–their ability to generate novel, surprising, and valuable ideas–is known to contribute to their learning outcomes, personal growth, and well-being. Creativity facilitates children’s problem solving, adaptability, self-expression and health (Carterette et al., 1994). Even though the benefits of creativity are widely recognized, classrooms are not able to sufficiently support children’s creative development. Gardner and Art, 1982 posited that children start to show creative abilities as early as preschool, and Smith and Carlsson (1983) found that the level of developmental maturity necessary for creative expression occurs around 5–6 yr of age. However, as they enter elementary school, children’s creativity slumps, especially around the fourth grade (Torrance, 1966, 1968; Claxton, 2005). As school curricula become more structured, children lose the aspect of creative play that is a significant part of kindergarten. To be successful in our AI-powered world, where mechanical and repetitive jobs are becoming automated, we must empower children to generate new artifacts and solve complex problems, which will require imaginative and novel thought.

In classrooms, social interaction plays a key role in children’s creative growth. Children learn creativity from teachers and peers who act as models for creative expression. They can scaffold children’s creativity through social interactions such as collaborating, posing challenges, asking questions, providing positive reinforcement, and generating ideas. Participating in collaborative tasks is one of the most effective external influencers of creativity (Kafai et al., 1995). In addition, classrooms today see increasing numbers of digital pedagogical tools and learning aids that have proven to be beneficial for cognitive learning due to their ability to personalize instruction for every student. Games and play-based learning approaches have been successful in fostering creative expression in children (Henriksen, 2006; Bowman et al., 2015). However, most educational technologies do not have the ability to foster social interaction with students. Exceptions include socially interactive AI agents such as conversational agents and social robots, which are highly effective in promoting learning and engagement (Chen et al., 2020). Previous work has demonstrated how social robots can influence children’s learning behaviors such as curiosity, growth mindset, and empathy through social emulation (Gordon et al., 2015; Park et al., 2017). Notably, the presence of a social robot can also affect adults’ creativity (Kahn et al., 2016; Alves-Oliveira et al., 2019). In this work, we explore how social robots can foster creativity in young children through collaborative, playful interactions.

Social robots are increasingly being used as learning peers and tutors (Adamson et al., 2021), and their unique ability to socially interact with children while being co-located has situated them well as creativity support tools (CST). Previous work has shown that robots are more effective than other mediums used for CSTs, such as screen-based interfaces (Kahn et al., 2016; Ali, 2019). We do not seek to compare robots to other mediums of CSTs in this work; rather, we intend to demonstrate the efficacy of creativity stimulating interactions designed for social robots. Furthermore, while social interaction is not a prerequisite for creativity, creativity literature informs us that social interactions with peers and tutors can foster creativity in children. Previous work has shown how situating the robot as a collaborative peer that offers ideas or helps with the creative process have benefitted creative expression (Louis and Peter, 2015; Rond et al., 2019). In this work, we explore whether a social robot’s capability for social interaction patterns can stimulate children’s creativity, while acknowledging that there are other stimulants of creativity. Learning from the effect of sociality on creativity in classrooms, we explore whether the effect can be replicated in pedagogical tools, specifically social robots. We suggest two interaction patterns in which intelligent embodied agents can help children think more creatively: *1*) creativity modeling, where the social robot models or demonstrates desired creative behaviors, and *2*) creativity scaffolding, where the robot offers scaffolding to the child in the form of asking reflective questions, validating novel ideas, and engaging in creative conflict. The robot used in this work is Jibo -- a child-friendly, tabletop, socially expressive robot.

We position our research in three playful and collaborative tasks, where children and the robot collaborate to create artifacts. These one-on-one interactions afford different forms of creative expression.

In the first two tasks, outlined in our previous research (Ali et al., 2019; Ali et al., 2020), we designed the behavior of the robot to artificially emulate human creativity:

1. The Droodle Creativity game, where children and the robot generate humorous titles for abstract images to express verbal creativity.
2. The Magic Draw game, where children and the robot co-create drawings on a tablet screen to express figural creativity.

In this work, we introduce the third task, where the robot scaffolds children’s creative thinking by asking questions, validating novel ideas, and engaging in creative conflict:

3. The WeDo Construction activity, where children and the robot co-create WeDo LEGO models to encourage constructional creativity.

We outline previous research, where we demonstrated that children can emulate a social robotic peer’s creative expression during collaborative gameplay. We ran two randomized controlled trials with 126 children: verbal creativity (*n* = 48) and figural creativity (*n* = 78) in the 5–10 yr old age group. Participants in the intervention group interacted with the robot exhibiting creative behaviors and participants in the control group interacted with the robot that did not exhibit these behaviors. We observed that children who interacted with the robot exhibiting high verbal creativity (in the Droodle Creativity game) and high figural creativity (in the MagicDraw game) exhibited higher verbal and figural creativity themselves. We then introduce our third study, where the robot offers creativity scaffolding behaviors in the WeDo Construction task. We ran a randomized controlled trial with 42 students in the 6–10 yr old age group. We observed that when the robot offered creativity scaffolding in the construction task, children expressed higher creativity.

In sum, we provide consistent evidence that the performance of creativity inducing behaviors by social robots can foster creativity in young children. Further, in all three studies, children in the high creative robot conditions perceived the robot as more creative and fun as compared to the low creative conditions. By showing that a social robot can successfully foster different kinds of creative expression, we are able to articulate more generalized social mechanisms that can be leveraged to support creativity in children. We contribute novel design guidelines and new methods for designing interactions with social agents that aim to promote creative thinking. With new developments in generative modeling techniques, robots can participate in several co-creative tasks with children, and can be leveraged as creativity support tools in a wide range of creative activities. We discuss implications for the field of HRI, digital creativity support tools, co-creative agents, and transformative games.

## BackGround

### Creativity

Creativity is often referred to as the ability to generate artifacts or ideas that are both novel and appropriate to the problem at hand (Carterette et al., 1994). Novelty is the ability to generate ideas that are different from one’s own ideas, and different from the group’s ideas. The appropriateness of a solution refers to solving problems using the least amount of time and resources. The definition of creativity has evolved from a function of the individual to an interaction between aptitude, environment, and process by which an individual produces a tangible product (Plucker et al., 2004). Depending on the nature of the task and the medium of creative expression, creativity presents itself in different forms; for example, figural creativity (drawing, painting, sketching) and verbal creativity (writing, storytelling, composition, discourse) (Guilford, 1957). In addition, construction (building, tinkering) is described as a form of creativity in which students can draw their own conclusions through creative experimentation and the creation of artifacts (Harel and Papert, 1991). A common means for fostering creative learning in classrooms is through construction and maker based activities, which we refer to as constructional creativity in this work.

Early creativity researchers defined creativity as the embodiment of thought in the form of external behavior, consisting of three characteristics: fluency, flexibility, and originality (Guilford, 1950). Fluency refers to the ability to generate several ideas, flexibility refers to the variation in themes between several generated ideas, and originality refers to the novelty of the ideas generated in comparison to those of the group’s. For the purpose of this work, we define the ability to generate ideas with greater fluency, flexibility and originality as creative thinking. Metrics of fluency, flexibility and originality are dependent on the choice of tasks made for each type of creativity. We also take divergent thinking into account as a component of creativity, and categorize activities that involve the creation of artifacts as activities that afford creativity.

### Extrinsic Factors Influencing Creativity

Researchers have identified several factors that may serve as “situational influences” of creativity: freedom, autonomy, good role models and resources, encouragement for originality, little criticism, and “norms in which innovation is prized and failure not fatal” (Amabile and Gryskiewicz, 1989; Witt and Beorkem, 1989). In our work, we utilize the following factors to design effective creative interactions.

#### Emulation

Emulation is described as “[when] children achieve common goals to those modelled, but do so by using idiosyncratic means that were never observed” (Bornstein and Bruner, 2014). Indeed, children are predisposed to social emulation (Yando et al., 1978), and learn from other creators in their environments, such as teachers and classmates, through mechanisms of social emulation (Whiten et al., 2009). Within classroom settings, researchers have suggested that the traditional educational model, which emphasizes rote problem solving, can be overcome by providing students with more diverse models of creativity to emulate (Root-Bernstein and Root-Bernstein, 2017). Social emulation may even be at the heart of innovation itself; one study showed that in a tower building task, children performed poorly at the task independently, but after observing one or two models building a tower, they were able to emulate the demonstrated elements and spontaneously recombine them, producing a novel tower of an optimal height (Subiaul and Stanton, 2020).

#### Social Interactions in the Classroom

The importance of the social environment to creativity is well researched (Kaufman and Sternberg, 2010). For children, the primary social environment is the classroom. Several factors that influence creativity, such as emulation, play, and collaboration, are heavily integrated into early education classroom curricula (Halverson and Sheridan, 2014; Kafai et al., 1995). Question-asking during creative activities stimulates their creativity (Zheng et al., 2013), and creative learning research outlines how game-based learning environments must facilitate reflective thinking (generating ideas and evaluating them) in order to foster creativity (Henriksen, 2006; Bowman, et al., 2015).

#### Collaboration

Creativity has typically been understood as an individualistic pursuit. However, it is now widely accepted that creativity stems from the confluence of diverse perspectives and ideas, and that the nature of collaboration stimulates creative problem solving (Kafai et al., 1995). For children in particular, several studies emphasize the importance of friendship and peership in fostering effective creative collaboration (MacDonald et al., 2000; Miell and MacDonald, 2000; Bass et al., 2008). The studies described in this work utilize a social robot as a peer in order to stimulate creative collaboration with children.

#### Play

Play-based learning tools and game-like activities have been repeatedly shown to promote creativity (Henriksen, 2006; Garaigordobil, 2006; Baggerly, 1999; Dansky, 1980; Howard-Jones et al., 2002; Mellou, 1995; Russ, 2003; Berretta and Privette, 1990). They are effective for teaching concepts to children since their entertainment value ensures higher engagement levels. Furthermore, several behaviors that constitute creativity can be promoted *via* gameplay behaviors, such as developing multiple solutions to a problem, generating novel and appropriate solutions, metacognition, question-asking, and cross-contextual thinking (Henriksen, 2006). Games designed to specifically alter players’ behaviors, attitudes, or knowledge during and after play are known as *transformational games*, which can be used as tools to support meaningful learning. Digital games in particular provide players with the opportunity to find many creative solutions within a singular play space (Bowman et al., 2015), especially in the case of well-known sandbox games such as Minecraft (Duncan, 2011). In our work, we utilize transformational digital games with game mechanics that allow for creative expression and creative problem-solving.

### Creativity Support Tools

Given the many extrinsic influencers of creativity, it is no surprise that HCI researchers have attempted to engineer creativity support tools (CSTs) “that empower users to be not only more productive but also more innovative” (Shneiderman et al., 2006). Since the framework’s proposal (Shneiderman, 2002), researchers have developed a wide range of CSTs. The vast majority of CSTs were built for digital devices, with the most common being a laptop or a personal computer (Frich et al., 2019). Previous work has demonstrated how creativity is also facilitate through analog toolkits such as Scratch Coding Cards (Scratch Team, 2017) and robotic construction kits such as Lego Mindstorms, Popbots, and Cozmo (Anki, 1999; Williams et al., 2019).

### Social Robots as CSTs

Despite creativity’s social nature, little work has been conducted on the benefit of utilizing social agents as CSTs. Previous work has demonstrated how verbal and non-verbal social robot behavior can serve to engage adults in a creative activity for longer and aid their own creative ideas (Kahn et al., 2016; Alves-Oliveira et al., 2019), and children will emulate a social robot’s expressed verbal and figural creativity, resulting in a higher level of creative expression (Ali et al., 2019; Ali et al., 2020). Alves-Oliveira et al. (2020) demonstrated how interacting with the robotic system YOLO when it displayed social and creative behaviors simulated children’s creative abilities. Robots with light patterns have also benefited children’s storytelling experiences (Ligthart et al., 2020). Another study showed how people spent more time creating music with drums while collaborating with the Mortimer robot (Louis and Peter, 2015). Rond et al. (2019) found that adult improve performers viewed a simple robot as a supportive teammate who positively inspired the scene’s direction. A majority of previous work utilized social robots as peers or partners. However, all mentioned works are specific to one creative task.

Social robotic agents are proven to be effective learning companions (Belpaeme et al., 2018), and children form relationships with them through social interactions (Westlund et al., 2018). There lies a unique opportunity in being able leverage these social and interactive agents as creativity fostering mechanisms for children. In this work, we demonstrate the efficacy of social robots as CSTs through three creative tasks that focus on three different kinds of creativity: verbal, figural and constructional. Further, we utilize game-based interactions since play is known to benefit creativity and it helps situate the robot as a collaborative playful peer. Like previous work, we situated the robot as a collaborative peer (Rond et al., 2019). Similar to Ali et al. (2019) and (Ali et al., 2021), we utilize the robot’s creativity demonstration as a creativity eliciting mechanism. Similar to Alves-Oliveira et al. (2019) and Kahn et al. (2016), we made use of the robot’s social verbal and non-verbal interactions during child-robot interaction. Through the three game interaction, and the robot assuming different social behaviors, we studied how creativity demonstration and creativity scaffolding through social interactions benefited children’s creative expression. We suggest interaction patterns of social robots specific to a computational learning setting that aim to foster creativity (described as Creativity Scaffolding interactions) that are generalizable to other creative tasks for children. Through this work, we aim to contribute to the literature of using social interactive agents as creativity support tools, through both their social interactions scaffolding children’s creativity, and their creativity demonstrations acting as a model for children to emulate.

## Robot Platforms

**FIGURE 1:**
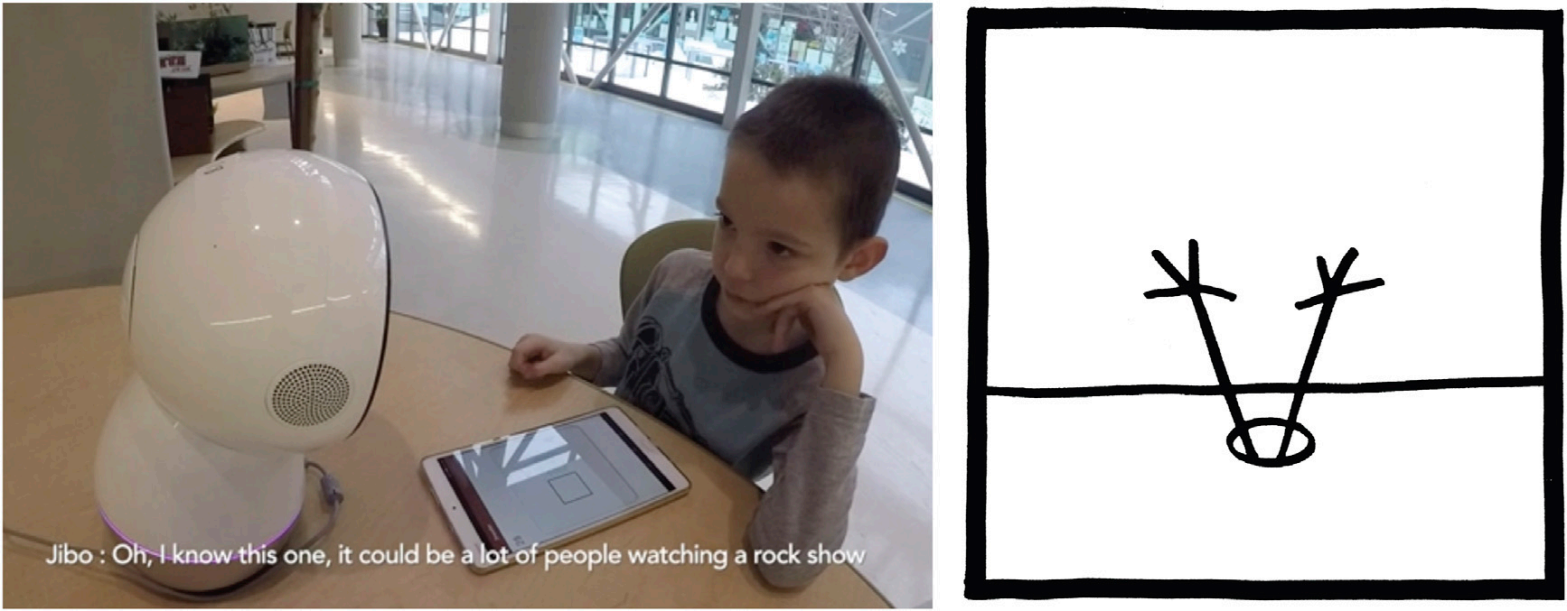
Interaction Scene. **(A)** A child is playing the Droodle Creativity Game on an Android Tablet with social robot Jibo. **(B)** Example of a Droodle Image. 10 Droodles were used in the Droodle Creativity Game (five per player).

For the three creativity activities, we used Jibo, a socially embodied robot, as our robotic platform (Jibo, 2015). Jibo is an expressive tabletop social robot (Figure 1) that can speak, respond to children’s speech, track faces, gaze toward the person, attend to sound and movement in its environment, and physically express emotion through its display and three degree-of-freedom body. Jibo can communicate with an Android tablet that serves as a shared drawing surface or to display information for the child. Prior to each study, the experimenters discussed with children that Jibo uses WiFi to see, talk, draw, and interact with objects displayed on the tablet, and that it doesn’t need physical hands to do so. Such discussion is important to building a believable experience for children that the robot knows what is displayed on the tablet and can draw on the tablet, too.

During the interaction, Jibo provides explanations and encouragement to the child, and expresses joy, curiosity, and pride. Between the high creative (C+) and low creative (C−) robot conditions, we carefully controlled the amount of verbal and non-verbal robot behaviors; however, the robot’s speech differed based on the study condition. In the C+ condition, the robot exhibited greater creativity; in the C− condition, it demonstrated substantially lower creativity.

We developed three game-based and interaction child-robot tasks which we introduce in the following sections.

## Experiment 1: Droodle Creativity Game

Previous work in HRI demonstrated how children emulate robots’ learning behaviors such as curiosity and growth mindset (Gordon et al., 2015; Park et al., 2017). Motivated from this literature, we explored whether this social emulation phenomenon extends to verbal creativity. Verbal creativity, defined as the ability to create verbal artifacts such as stories, prose and poetry has three indicators: fluency, or the ability to produce a large number of ideas; flexibility, or the aptitude for changing from one approach to another or from one line of thinking to another; and originality, or the capacity for bringing new ideas or solutions that are far from obvious, common, or established. Development of verbal creativity in children is pivotal to their learning, writing and thinking skills and helps them reflect their feelings, emotions, opinions, reactions, and notions to others (Shorofat, 2007; Rababah et al., 2017). In this work we explore whether a social robot’s creative verbal expression is emulated by children, for which we created the Droodle Creativity game (Ali et al., 2019), inspired by the Droodle Creativity Task, a verbal creativity task that draws upon people’s ability to creatively use language to describe an abstract image or figure known as a *droodle* (Kahn et al., 2005). The Droodle Creativity Task has been previously validated as a means to measure people’s verbal creativity, and is based on the cartoon book Droodles by Roger Price (1982), thus making it appropriate for a children’s game. The Droodle game encourages children to think creatively, express their thoughts and encourage humor.

### Game Design

In the Droodle Creativity Game, two players take turns to generate Droodle titles (Figure 1). The active player is presented with droodles on a tablet screen and they come up with droodle title(s) in 30 s. Then the turn shifts to the other player until each player has played five turns each. The Droodle Task coding system, developed by Kahn et al. (2005), provides a metric for ranking the titles as “non-droodle,” “low-,” “medium-,” or “high-droodle” based on the participant’s initial reaction, pattern matching to the image in question, and reasoning for providing such an answer. Droodles used in our study were taken from *Droodles: The Classic Collection* (Price and Lovka, 2000) which also includes a library of droodle titles.

### Interaction Scenario

When the interaction starts, Jibo explains to the child how the Droodle task works and engages the child in a practice round. When the child generates a creative droodle title, the robot praises the child by using phrases such as, “Great job,” “I would not have thought of that,” or “You are doing great.” When the robot is “thinking” of a Droodle idea, it expresses curiosity through questioning sounds, swaying movements, and looking upwards.

### Experiment Design

#### Participants

We recruited 51 subjects in the 5–10 yr age range as a part of the Somerville after-school activities program at the public schools in Somerville, MA. All students had basic knowledge of robotics and artificial intelligence taught to them as a part of another module of the after-school program. Three students were excluded due to technological malfunctions or a rudimentary understanding of English.

All participants and their guardians signed an informed assent and consent form to participate in the study and permit us to collect demographic, assessment, audio and video data. The recruitment materials, study protocol, and data collection protocol were reviewed and approved by the Institute Research Board at Massachusetts Institute of Technology.

#### Pre-test

All students completed the Torrance Test of Creative Thinking (TTCT) assessment as a part of the pre-test activity (Torrance, 1968). The TTCT is a paper-based evaluation that consists of two sets of assessment activities: a verbal creativity test and a figural creativity test. The purpose of conducting the TTCT before the study was to drive a quasi-random assignment into groups such that their creativity scores are counterbalanced across the two conditions, described in the following section.

#### Study Conditions

Forty-eight participants were divided into two study condition groups, one that interacted with the high creative robot (C+) and one that interacted with the low creative robot (C−). The groups were divided such that the participants in the two groups were balanced in terms of their mean and standard deviations of TTCT scores (C+: 42.16 ± 7.17; C−: 40.66 ± 6.01), age (C+: 7.78 ± 1.92; C−: 8.38 ± 1.85), and gender (C+: F = 9, M = 15; C−: F = 13, M = 11).

The robot exhibits high or low creativity during gameplay depending on the study condition (Table 1). We use Boden’s framework of creativity to design creative behaviors in gameplay (Boden, 2004), and Kahn et al.’s Droodle Task Coding system (Kahn et al., 2005) to determine the creativity of Droodle titles.

**TABLE 1:** A gameplay comparison of the high creative and low creative study conditions.

	Fluency	Novelty	Value
High creative robot (C+)	Robot generated four to five ideas per Droodle	Robot explored three or more different themes	Robot picks Droodle titles that are tagged medium/high in creativity
Low creative robot (C−)	Robot generated one to two ideas per Droodle	Robot explored one to three different themes	Robot picks Droodle titles that are tagged low/medium in creativity (e.g. the literal description of an image)

#### Hypotheses

H1: Participants interacting with the high creative robot (C+) generate a larger *number* of ideas than participants interacting with the low creative robot (C−).
H2: Participants interacting with the high creative robot (C+) explore more *themes* of ideas than participants interacting with the low creative robot (C−).
H3: Participants interacting with the high creative robot (C+) generate more *creative* ideas than participants interacting with the low creative condition (C−).

#### Data Collection and Measures

Children’s speech and video data was recorded. We used Google Cloud’s Speech API (Google, 2020), as well as manual transcribing by three researchers blind to the study to transcribe children’s phrases. We used the TTCT to assess children’s verbal and figural creativity prior to all study interactions, and to divide them into balanced study groups.

We measured participants’ creativity in three parts:

• *Fluency.* The number of ideas that the participants generated.
• *Novelty.* The number of unique themes explored through the ideas. Each idea is associated with theme tags, which include all concepts and keywords included in the idea.
• *Value.* The droodle creativity scores of the ideas generated. Droodles are graded on a scale from 0 to 3, mapping to non-droodle, low-droodle, medium-droodle, and high-droodle respectively (Kahn et al., 2005).

For instance, one participant came up with the following ideas for the droodle image in round 1 (Figure 1): “*It’s peppa pig*”; “*It’s peppa pig’s hands*”; and “*It’s frog hands*.” This would be analyzed as: Number of ideas (fluency) = 3; Unique themes (novelty) = “*peppa pig*,” “*hands*,” “*frogs*”; Droodle scores (value): 2, 3, 2.

#### Results

We calculated numerical values for each of the three creativity measures, then further determined the mean and standard deviation of the *Novelty* and *Value* scores for every Droodle image for each participant. For instance, if a participant generated three ideas for Droodle #1, the *Novelty* and *Value* would be the mean score of the three individual *Novelty* and *Value* scores. We then conducted unpaired T-tests between the high creative and low creative study participants to determine any between group differences for each of the three metrics.

H1: Participants interacting with the high creative robot (C+) generate a larger *number* of ideas than participants interacting with the low creative robot (C−).

To test our first hypothesis, we analyzed the number of ideas generated by the participants in the two study conditions. We observed that participants who interacted with the robot expressing high levels of creativity (C+) generated significantly more ideas (*t*(29) = 1.699, *p* < 0.01**) compared to the participants who interacted with the robot expressing low levels of creativity (C−) (Table 2).

H2: Participants interacting with the high creative robot (C+) explore more *themes* of ideas than participants interacting with the low creative robot (C−).

**TABLE 2:** Droodle Creativity game *t*-test results per condition for each study hypothesis.

SG	Ideas generated (H1)	Themes explored (H2)	Creativity scores per Droodle (H3)
**C+** (*n* = 24)	3.325 ± 1.16	4.983 ± 1.25	1.73 ± 0.21
**C**− (*n* = 24)	2.417 ± 0.96	3.842 ± 1.66	1.532 ± 0.25
*p*	*t*(29) = 1.699, *p =* 0.006	*t*(29) = 1.699, *p =* 0.010	*t*(29) = 1.699, *p =* <0.015

Participants in the C+ condition generated more ideas, explored more themes, and overall received higher creativity scores than participants in the C− condition.

To understand the novelty of the themes that participants generated, we used the Rapid Automatic Keyword Extraction algorithm (Rake NLTK), a natural language processing library, to analyze the themes explored in each title (Mumford, 2001). We observed that participants in the C+ condition explored significantly more overall unique themes (*t*(29) = 1.699, *p* < 0.01**) as compared to the participants who interacted with the robot expressing low levels of creativity (Table 2).

H3: Participants interacting with the high creative robot (C+) generate more *creative* ideas than participants interacting with the low creative condition (C−).

Three coders blind to the study conditions were trained using the Droodle creativity coding scheme. They then coded all Droodle titles generated by the participants as “non-,” “low-,” “medium-“ and “high-droodle.” To determine inter-rater reliability between researchers, Cohen’s kappa (Hallgren, 2012) was calculated using 67% of the coded transcripts coded independently by a team member after an initial coding by other two coders. Cohen’s kappa was 0.82, which is within the range for substantial agreement considered acceptable for inter-rater reliability.

**FIGURE 2:**
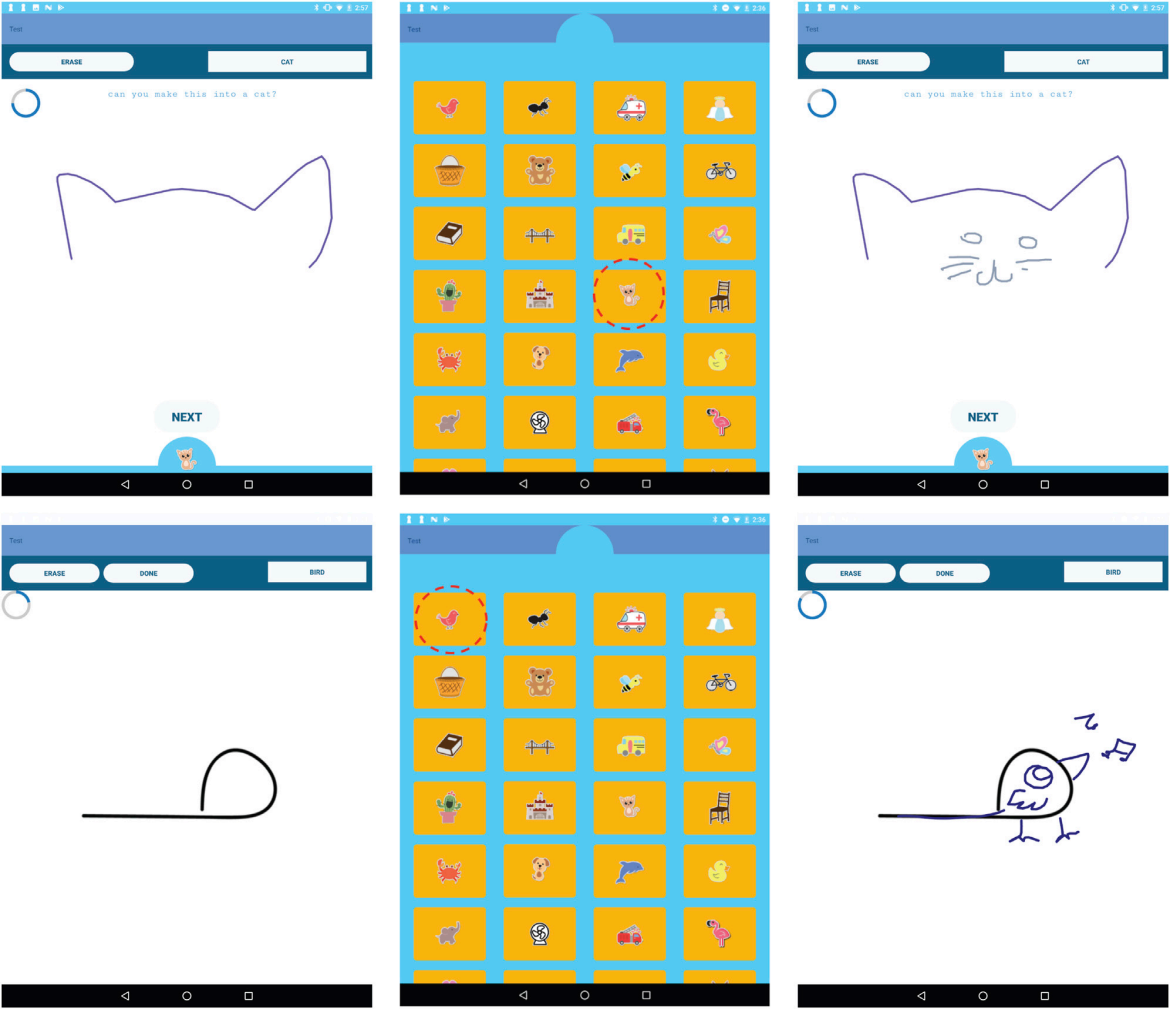
Interface screenshots of the Magic Draw game explaining the child-robot gameplay. **(A)** The child draws a starting prompt (cat ears). **(B)** The child selects a target category (cat). **(C)** The robot tries to convert the starting prompt (circle) into the target category (cat). **(D)** Players switch turns and the robot gives the child a starting prompt. **(E)** The robot selects the category (bird). **(F)** The child converts the starting prompt to a bird. (order: left to right starting top-left).

An overall analysis of creativity scores for every idea revealed that participants in the creative condition scored significantly higher in creativity score per title than participants in the low creative condition (*t*(29) = 1.699, *p <* 0.01**) (Figure 2).

## Experiment 2: MagicDraw

Similar to verbal creativity, we aimed to explore whether children also emulate a robots’ expressed figural creativity. Generative models such as GANs make it possible for AI models to generate creative drawings (Ha and Eck, 2017; Ge et al., 2020). Collaboration with both humans and digital interfaces has also been known to benefit figural creativity (Kim et al., 2016). Collaborative digital CSTs have been constantly evolving to support the creativity needs of people, and have the ability to contribute to the drawing itself. In human-human creative collaborations, creators can socially interact with one another, provide feedback and comment on their drawings, an interaction style that is lost in digital CSTs. In this work, we explore whether collaborating with a robot that also interacts with the creators socially, in a figural co-creation activity benefits children’s creativity. We explore whether children emulate the robot’s expressed figural creativity through a co-doodling task.

### Game Design

**FIGURE 3:**
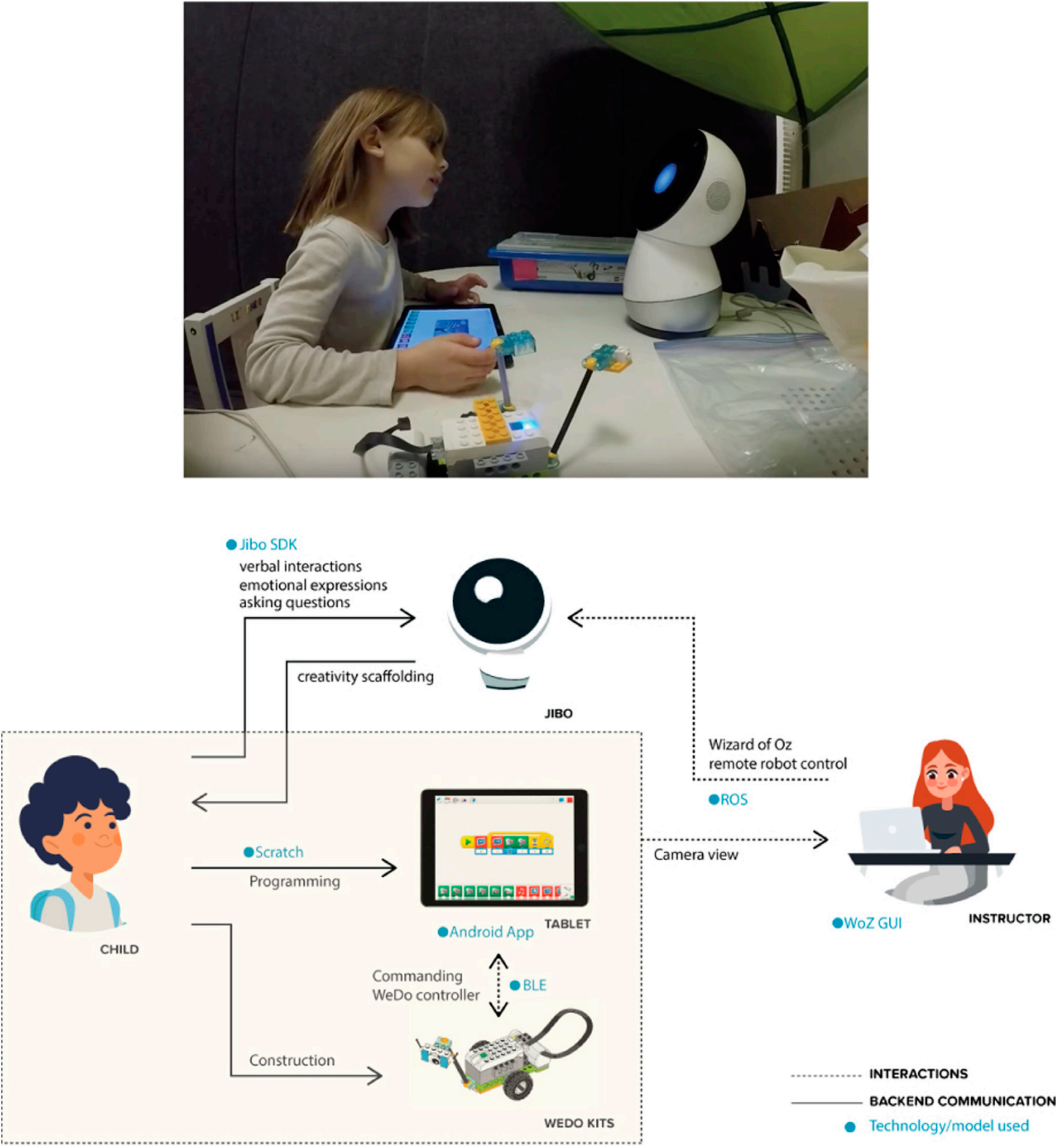
(top) **(A)** Child constructing models with Jibo. (bottom) **(B)** Model of interaction for the WeDo construction game where the robot provides creativity scaffolding.

To investigate whether children model a social robot’s figural creativity, we designed the MagicDraw game, which involves a collaborative drawing interaction on an Android tablet between the child and the robot (Ali et al., 2020). The gameplay requires one player to start a drawing with a stroke, and the other player completing the drawing. After the drawing is complete, the players switch turns. When it is the robot’s turn to complete the drawing, we utilize the Sketch-RNN model (Ha and Eck, 2017), which generates drawing strokes to convert a starting stroke into a meaningful illustration (Figure 3).

### Interaction Scenario

The collaborative figural activity utilized Jibo for similar reasons to the collaborative verbal activity, and its interactions were designed to evoke an autonomous “artistic” peer, collaboratively creating drawings with the child. Even though Jibo does not have appendages, it conveys interest and intent by looking at the tablet while it “draws,” and vocalizes relevant phrases, such as “OK, a cat. I think I can make that drawing into a cat.” “Here I go!,” or “Watch me convert your doodle into a cat.” The robot can also ask the child for feedback, e.g. “What do you think about my drawing?,” increasing the credulity of the interaction. We verified in the post-test that children perceived Jibo was drawing on the tablet with them. Similar to the Droodle Creativity Game, subjects and Jibo played the figural creativity game by taking turns on an Android tablet.

### Experiment Design

#### Participants

We recruited 78 children in the 5–10 yr age range as a part of the Somerville after-school activities program at the public schools in Somerville, MA. Eleven students in the figural creativity study were excluded due to incomplete data collections or network errors.

#### Pre-test

All students completed the TTCT assessment as a part of the pretest activity. As with the prior study, the purpose of conducting the TTCT was to drive a quasi-random assignment into groups such that their creativity scores are counterbalanced across the two conditions, described in the following section.

#### Study Conditions

Participants were divided into two study condition groups: one that interacted with the high creative robot (C+) and one that interacted with the low creative robot (C−). The groups were divided such that the participants in the two groups were balanced in terms of their TTCT scores (C+: 43.33 ± 6.30; C−: 42.91 ± 5.16), age (C+: 7.89 ± 1.91; C−: 7.09 ± 1.96) and gender (C+: F = 14, M = 23; C−: F = 20, M = 21).

The robot in the C+ condition produced more creative drawings as defined by the metrics of the Test of Creative Thinking - Drawing production (TCT-DP) -- a figural creativity test (Urban, 2005). During the robot’s turn, we adjusted the drawing model to reduce the randomness in drawing. We kept the speed of drawing to the default speed (60 fps). The robot always drew true to the selected category. This led to higher quality drawings with a better model match to the category that the child selects. The length and number of interactions were controlled for across the two conditions. We validate this hypothesis of these drawings being rated as more creative in the following section.

In the low creative robot condition, the robot system was configured to produce less creative drawings as measured by the TCT-DP figural test parameters. We adjusted the generative model to increase the randomness of the drawing, thereby producing lower quality drawings with a lower model match to the category that the child selects. Further, we adjusted the frame rate of rendering to 30 frames per second to generate the drawings more slowly. We also made the model periodically select an incorrect category to make the drawing not match the selected theme.

#### Hypotheses

H1: The drawing model produces more creative drawings in the C+ condition than in the C− condition.
H2: Children who played the Magic Draw with the high creative Jibo (C+) will exhibit higher levels of creativity in their own drawings than children that play with the low creative Jibo (C−).

#### Data Collection and Measures

The MagicDraw application logged the drawings done by the child from all three rounds, the drawings done by the robot in all three rounds, and the time taken for each drawing onto a log file downloaded to the Android tablet. We also used an overhead GoPro camera to take a birds-eye video of the interaction, as well as for recording audio and participants’ post-test interviews.

To assess figural creativity from children’s drawings in the MagicDraw interaction, we used the Test of Creative Thinking - Drawing Production (Urban, 2005). Three coders blind to the study’s hypothesis and the participants’ study condition reviewed the drawings and rated them. These scores were then used for calculating the TCT-DP measures of the drawings. Some participants did not make any drawings, and some drawings were not saved due to network errors.

#### Results

We conducted unpaired t-tests comparing the creativity scores of drawings generated by participants in the control condition (C−) and the experimental condition (C+), as measured by the TCT-DP test.

H1: The robot’s drawing model produces more creative drawings in the C+ condition than in the C− condition.

To test this hypothesis, we compared the TCT-DP scores of the robot drawings generated by the creative model and by the low creative model. An unpaired *t*-test showed that the model type had a significant effect (p < 0.01**) on the generated drawing’s corresponding creativity score (Table 3). This dataset was notably smaller than the children’s drawing dataset since we did not collect all of the drawings generated by the model. This significant difference helped establish that the creative model was indeed generating drawings that were more creative than the low creative model. Hence, manipulating certain parameters of the model led to a change in the drawing’s creativity.

H2: Children who played the Magic Draw with the high creative Jibo (C+) will exhibit higher levels of creativity in their own drawings than children that play with the low creative Jibo (C−).

**TABLE 3:** MAGICDRAW figural creativity game one-way ANOVA results per condition for each study hypothesis.

Model Condition	Robot Drawing TCT-DP scores (H1)	Children’s Drawing TCT-DP scores (H2)
High creative model (C+) (*n* = 37)	28.91 ± 6.69	42.27 ± 14.30
Low creative model (C−) (*n* = 41)	20.33 ± 7.86	32.88 ± 9.64
Result (unpaired t-test)	*t*(26) = 1.60, *p* = 0.0064	*t*(56) = 1.67, *p* = 0.0023

One way ANOVA tests revealed that the study condition has a significant effect on participants’ figural creativity.

**FIGURE 4:**
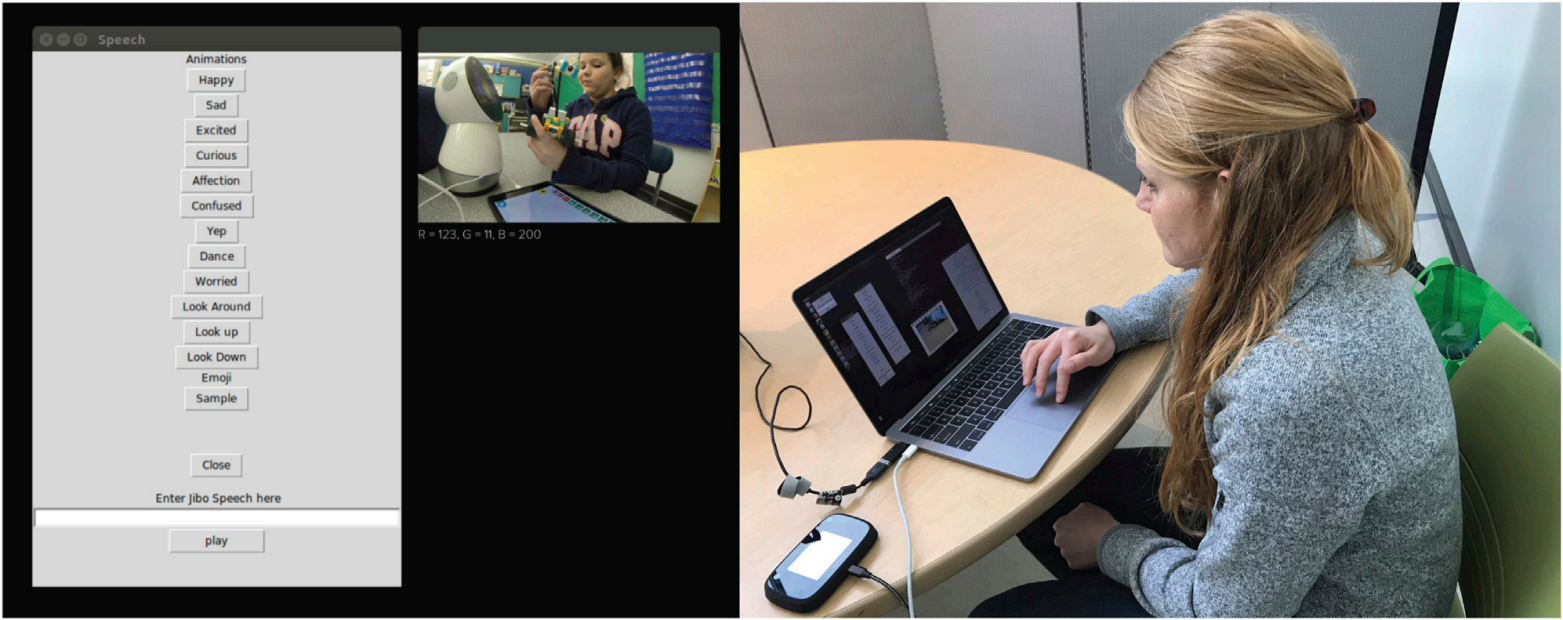
**(A)** Version 1 of the Jibo control interface for generating Jibo speech commands and emotional expressions. **(B)** A teacher using the remote control interface to control the robot.

To test this hypothesis, we compared the TCT-DP scores of all participants in all the conditions. An unpaired *t*-test test revealed that the study condition had a significant effect on children’s figural creativity, and showed a significant difference between the High creative robot (C+) and Low creative robot (C−) (*p* < 0.01**) (Figure 4). We could hence validate our second hypothesis.

## Experiment 3: WeDo Construction Task

Physical construction, or the ability to make new artifacts by combining other artifacts, is a key indicator of children’s creativity. Furthermore, unique ways of combining and using the creations also indicates divergent thinking. Physical construction kits facilitate playful learning, open-ended making and creativity, and often creates co-creation space with others (Alimisis, 2013). Robotic toolkits and environments have been successfully leveraged to afford construction by children (Mioduser and Levy, 2010). When children construct with others, their creativity is scaffolded by other children acting as models to emulate, providing ideas, brainstorming, challenging and asking questions. In this experiment, we explore whether this social support can be offered by a social robotic collaborator in a construction activity.

To afford constructional creative expression through making, we designed a third activity in which children and Jibo collaborate building models using the LEGO Education WeDo 2.0 Core Set (2020). The set consists of LEGO bricks and electronics that can be programmed using a visual programming interface on a tablet application with the aim of introducing children to computational thinking and engineering principles in a fun and engaging way.

**FIGURE 5:**
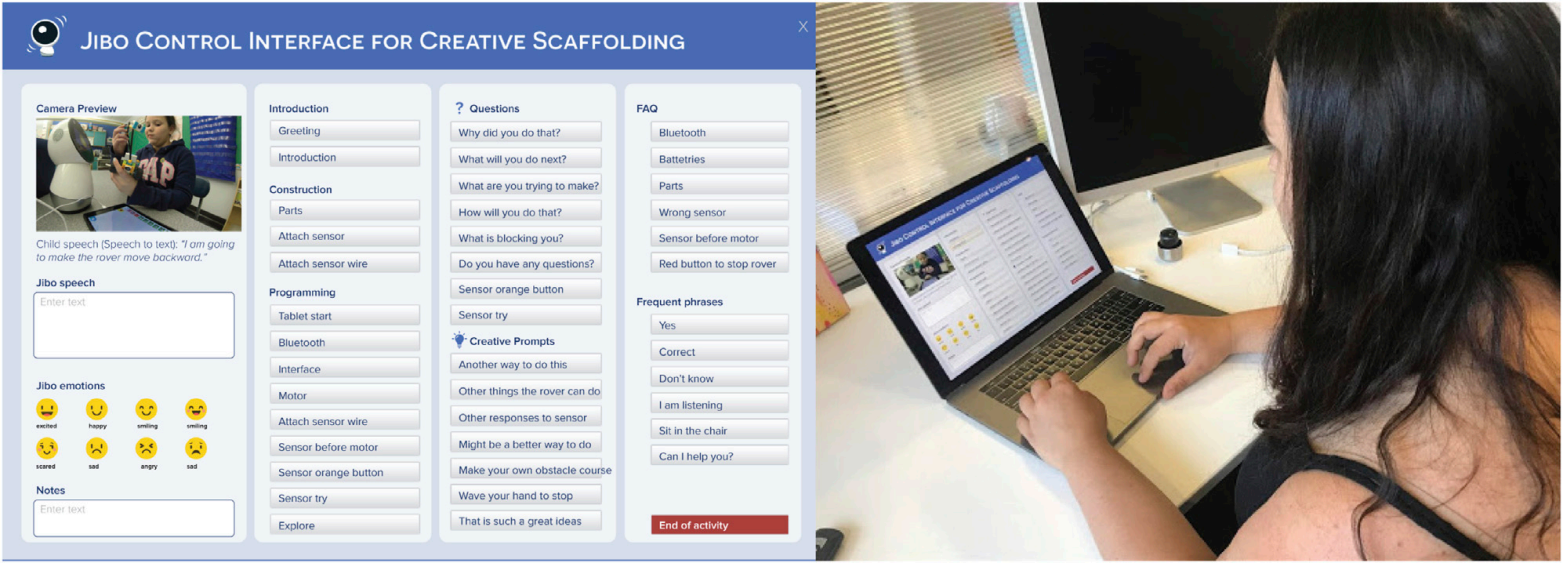
**(A)** The Jibo control interface. The questions, creative prompts, and positive reinforcement prompts only appeared for the instructors in C+ condition. **(B)** A teacher using the remote control interface to control the robot.

The interaction involved children making construction projects using the WeDo 2.0 kit in the presence of a social robot, which assumed the role of a tutor and provided scaffolding to the child. In order to get acquainted with the programming interface, children were first guided by the robot to build a rover using LEGO blocks, and to program the rover such that it could detect obstacles and respond to their commands using the WeDo Android tablet application. Children could utilize WeDo 2.0 standard construction kit items, including a Bluetooth enabled controller, LEGO bricks, motors and supporting construction materials and motion sensors. This guided activity was conducted through a step-by-step verbal exchange between the child and the robot and lasted for 6 min, with the robot taking the instructor role (Figure 5). The activity introduced the child to sequential commands, condition statements, delays, and loops. Then, children were given 20 min for free play, where they could explore different functions of the WeDo app, add new LEGO blocks, and make their models perform new actions. The idea generation process was guided by both the child and the robot. The role of the robot was to scaffold the child’s creative learning through verbal and non-verbal behaviors. Throughout the interaction, children could ask the robot questions and receive dynamic troubleshooting guidance. The robot also engaged in active creativity scaffolding which involved asking the child reflective questions, challenging their ideas and assumptions, and suggesting alternate ideas for creations with the rover. The robot also provided feedback and positive affirmation after children generated new ideas.

While the Droodle Creativity game and MagicDraw game utilized a fully autonomous Jibo interaction, in this activity, the robot was controlled by a human instructor using a dynamic and predictive Graphical User Interface (GUI) on the desktop to provide quality scaffolding at the right time and also to respond to the child appropriately. This GUI controller was iteratively co-designed in tandem with the instructors, with the goal of assisting them in providing creativity scaffolding to the children. This iterative process of designing the GUI is outlined in Section 3.4.2. The desktop GUI application communicated with the robot using Robot Operating System (ROS, 2021). Children programmed the WeDo controllers using an Android application on a tablet screen. Figure 5 illustrates the system components and communications between them.

### Interaction Scenario

#### Introduction

Jibo guides the activity with the child, starting with a self-introduction and then engaging in a short ice-breaker activity. Jibo begins by looking at the child and saying, “Hi. My name is Jibo. What is your name?.” When the child responds with their name, Jibo replies with an affectionate expression while saying, “It is so nice to meet you. My favorite activity is to do my favorite dance! What activities do you like?”

Jibo explains the activity to the child: “Today, we will be programming this rover robot to do cool things. [looks down at the tablet] Are you ready to begin?” When the child says “yes,” Jibo responds with excitement, “Let’s go!”

#### Child-robot Co-play

Jibo then begins a step-by-step guided activity to help the children learn how to program a rover with the LEGO WeDo kit. This activity ensures that children understand how to use the WeDo construction kit. After this guided activity, children are free to explore and build new models with the WeDo kit. Jibo provides creativity scaffolding to help children generate novel ideas by using verbal phrases like “Can you think of another way to do this?”

#### Robot Interactions

Jibo acts as an instructor by: *1*) assisting children in learning how to use the WeDo construction kit, and *2*) scaffolding children’s creativity while they construct models. While the tone of interaction is collaborative -- for instance, Jibo says, “Today, we will be building a rover together” -- Jibo primarily takes on the role of a tutor that is helping children to create something. Jibo interacts with children through speech prompts and emotional expressions. Jibo’s behaviors are remote controlled by a human instructor in a Wizard-of-Oz (WoZ) manner.

Actions for creativity scaffolding are inspired by how human instructors and peers scaffold children and enable them to be more creative by asking reflective questions, generating multiple diverse ideas, challenging assumptions, providing feedback, and appreciating the value of the child’s ideas. Creativity and divergent thinking literature elaborates on how asking reflective questions, presenting challenges, and positive reinforcement fosters creativity in children and adults (Kafai et al., 1995; Halverson and Sheridan, 2014). Collaboration with peers and tutors is also beneficial for creative thinking (Rojas-Drummond, et al., 2008). In this activity, instructors use a remote control GUI that has preset suggestions for prompts. We gathered the prompts from collecting and categorizing interaction data from human instructors scaffolding children’s creativity using an open-ended WoZ interface. The design of the interface is described in detail in the following section.

### WoZ Creativity Scaffolding Interface Design

**FIGURE 6:**
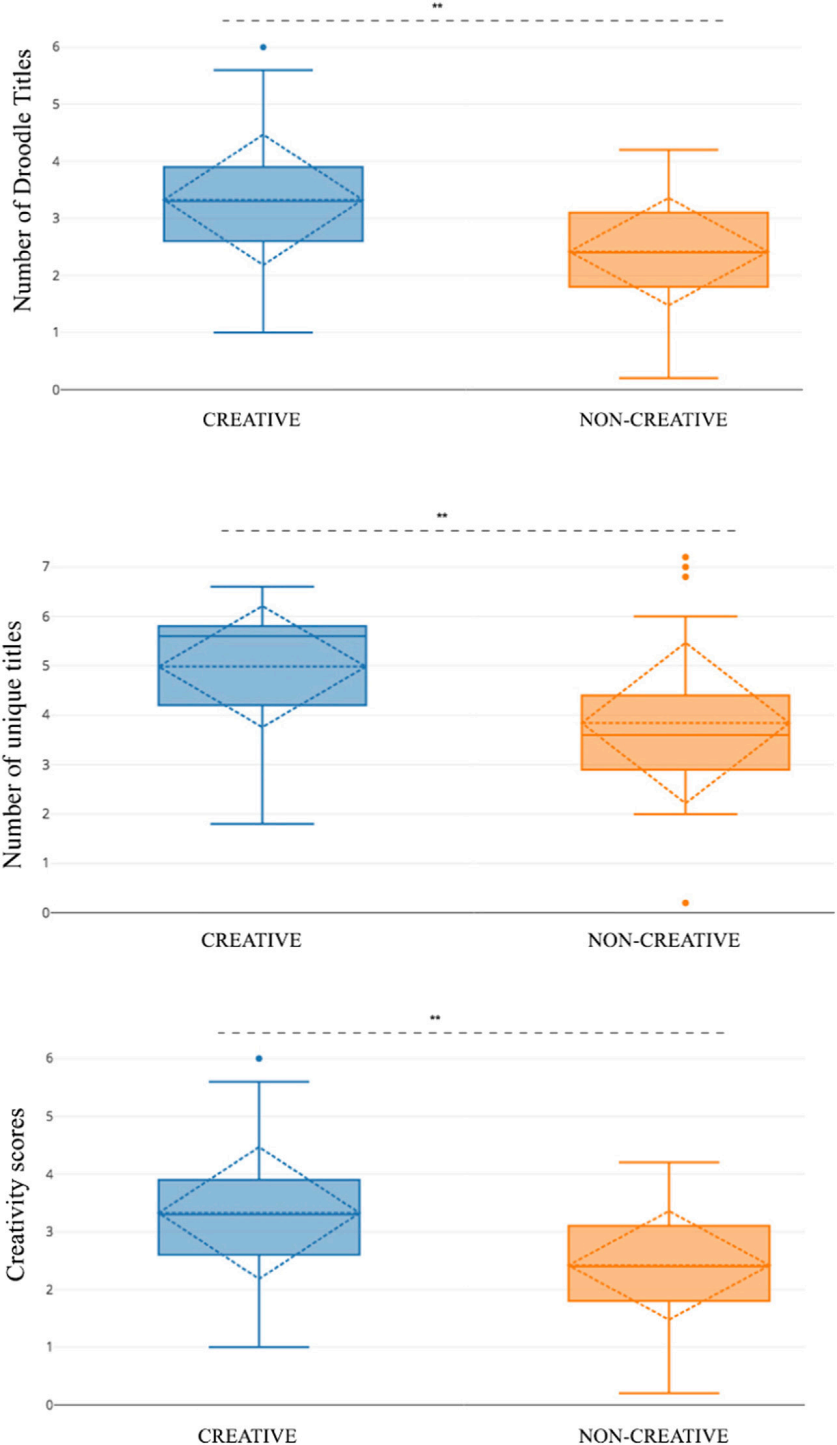
Participants in the C+ condition group generated significantly more Droodle titles, more unique titles and highly creative titles as compared to the C− group.

The goal of designing a robot control interface was to provide instructors with a user-friendly tool to remotely control Jibo while enabling them to provide creativity scaffolding to children. First, three instructors were given a fully flexible desktop interface which contained a text box where they could freely create dialogues and buttons to choose from preset animations for Jibo, while overseeing the interaction using a birds-eye camera view (Figure 6). All instructors were trained collectively to understand the task and the WeDo construction kits. The instructors were told that their task was to guide the children to build a simple rover, and then to assist children in thinking creatively about building other WeDo models. Further, all instructors were given a detailed protocol guide for the graphical interface to control Jibo. We evaluated the interface with three instructors and eight participants (6–10 yr old).

An affinity diagramming method was used to categorize all of the prompts that were used by the instructors. And resulted in the following categories:

• Instructions: Construction and programming instructions with the goal of teaching children how to use the WeDo construction kits and build their rover. Instructors tended to use the same language of instruction that was provided to them in the protocol.
• Questions: Reflective questions that the instructors asked children. For example, “Can you tell me why you did that (last action)?,” “Can you think of another way to do that (last action)?,” or “How will you do that?”
• Creativity prompts: All prompts that were not direct instructions to children but were focused towards helping them come up with creative ideas. These included new ideas and challenges. Prompts included “Can you think of another way to use that block?” or “What else can you make with the same blocks?”
• Feedback: All responses to children’s actions. These were mostly positive feedback, such as, “Good job,” but also involved encouragement prompts such as “Let’s try again.”
• Frequently asked questions (FAQ): There were times when children asked the robot questions to help them troubleshoot problems. Some patterns that arose were difficulty connecting the rover to Bluetooth, or not being able to find a part. The responses were first grouped by topics such as “Bluetooth,” or “Missing parts” and then all of the instructors’ responses to these questions were grouped under FAQ.

**FIGURE 7:**
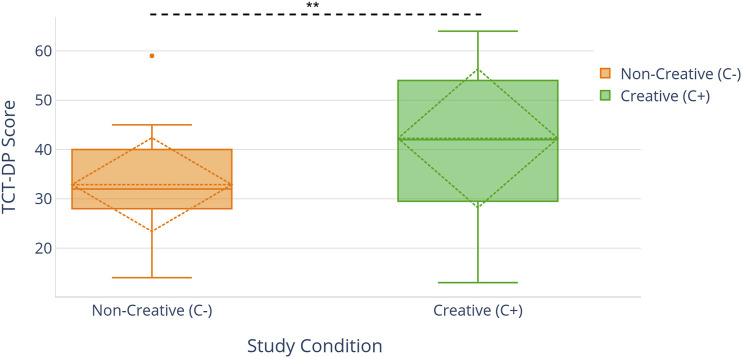
Participants who interacted with the high creative robot scored significantly higher on the TCT-DP test compared to the low creative robot condition (H2).

We then provided teachers with a more structured GUI for interaction, where frequently used speech prompts were made into buttons in order to reduce time delays, and organized by their categories, as shown in Figure 7. Instructors could also input custom speech if needed. We evaluated the interface using a think-aloud evaluation protocol (Ericsson and Simon, 1980), where the instructors spoke about what actions they want to perform, how they use the interface to perform it, and what the interface does not allow them to do. At the end of the interaction, the instructors were asked the following questions:

• What worked in the interface to help you give instructions and scaffold for creativity?
• What did not work in the interface?
• Were there parts of the interface that you did not understand the functionality of?
• What will you change in the interface to better suit your needs?

Based on their interactions with the interface and feedback post interaction, we iterated on the interface design. For instance, an instructor expressed that the interface was too cluttered, and short “clues” to the prompts would be preferable compared to the entire prompt for ease of use. Another instructor expressed how getting a sense of time elapsed on the screen can better prepare them in planning the activity. We stopped the iteration loop when the instructors reported that the interface presets were sufficient for their needs barring some outlier interactions. The final set of creativity scaffolding prompts used in the interface buttons are listed below.

Reflective Questions:

• Can you tell me why you did that?
• What will you be doing next?
• What are you trying to make?
• How are you going to do that?
• What are the materials you would be needing for that?
• Do you have any questions about this?
• Is that the best way to do that?

Creative Prompts (Ideas and Challenges):

• What are some other things you can make the rover do?
• What else can you make the rover do when an object is near? Can you make it have a different output?
• What are some other uses for the [motor/sensor]?
• Let’s think of some fun uses of the rover.
• There might be a better way to program that.
• I have an idea!
• Let’s try to make an obstacle for the rover’s sensor to detect. You can use LEGO blocks to make an obstacle.
• Let’s try to make the rover move when you wave your hand in front of the sensor, and stop when you wave your hand again?

Positive Reinforcement:

• That is such a great idea! Good job.
• You think of some really cool use of the robot.
• Well done. That was so creative.
• Great job!
• You are doing so well.
• I would not have thought of that. Good going.

We conducted 20 playtests, logged instructor’s interactions with the GUI, and coded them with: *1*) the previous GUI interaction, and 2) the child’s actions that led to the interaction. We then calculated the probability of each GUI interaction following each child action, or preceding GUI interaction. For instance, the child connecting the sensor to the rover body had the highest probability to be followed by the prompt “Instruction: try out the sensor by waving your hand” (Prob. = 0.58). We used these probabilities to predict what the instructor’s next interaction with the GUI would be based on the child’s actions and the instructor’s previous GUI interaction. We developed a dynamic predictive suggestions feature where the interface would prompt the instructor with GUI elements to use when the child performed a certain action. Instructors could choose to use the predicted prompt or create a new one.

### Experiment Design

#### Participants

A total of 43 participants in the 6–10-yr-old age group were recruited for our third study (19 female, 24 male). All students completed the TTCT as a part of the pretest activity. The average age of the participants was 8.11 (S.D. = 1.68). The subjects were recruited as part of the Somerville after-school activities program at the public schools in Somerville, MA. All participants and their guardians signed a consent form to participate and for audio and video data collection. Three adult instructors were also recruited for the study. All instructors were given preliminary training of WeDo construction kits, the programming interface, and a study protocol.

#### Pre-test

Participants were administered the first part of the verbal and figural module of the TTCT.

#### Study Conditions

Participants were divided into two study-condition groups: one that interacted with the robot offering creativity scaffolding (C+ condition) and one that interacted with the robot not offering creativity scaffolding (C− condition). The groups were divided such that the participants in the two groups were counterbalanced in terms of their mean and standard deviations of TTCT scores, age and gender (Table 4).

**TABLE 4:** Study groups for WeDo Construction task.

Study Groups	*n*	TTCT scores	Gender	Age
High creative (C+)	23	42.16 ± 7.17	F = 11 M = 12	8.3 ± 1.57
Low creative (C−)	20	40.66 ± 6.01	F = 8 M = 12	7.65 ± 1.85

43 participants were divided in balanced groups based on TTCT scores, gender and age.

In both study conditions, the robot was controlled by a human instructor (blind to the study condition) using a WoZ desktop interface (Figure 7). In both conditions, instructors were instructed to start with providing the same set of basic instructions to help the child build and program a rover model that incorporates a sensor and a motor, after which they are left to explore and make their own models. Instructors sent construction commands provided in the WoZ interface after the child completed the previous instruction, as was indicated by the live camera field. Instructors in the creativity scaffolding condition (C+) were instructed to ask reflective questions, challenge the participants, and collaboratively ideate with them. In order to facilitate this scaffolding, they are equipped with the creativity scaffolding interface, which in addition to the construction instructions, also consisted of questions, creative prompts and positive reinforcement prompts (as outlined in Section 3.4.2). In contrast, in the C− condition, the robot prompted the child to explore and make new things in the beginning of the free exploration period, and the instructors were instructed to only participate to answer questions beyond that. In the C− condition, the scaffolding interface also lacked the questions, creative prompts and positive reinforcement prompts. The three instructors were paired with an equal number of C+ and C− condition participants to control for experimenter bias.

#### Hypothesis for the Creativity Scaffolding Study

In order to understand the effect of creativity scaffolding on children’s creativity, we hypothesize that participants who interacted with the robot offering creativity scaffolding exhibit higher levels of creativity in the WeDo construction task. We divide our hypothesis in these parts derived from the three ways of assessing creativity behaviors during the task:

H1: Participants who interact with the robot offering creativity scaffolding (C+) generate a greater number of ideas and use cases for the rover than those who interact with the robot without creativity scaffolding (C−).
H2: Participants who interact with the robot offering creativity scaffolding (C+) use a higher number of new programming blocks (excluding the blocks used in the instructions) than those who interact with the robot without creativity scaffolding (C−).
H3: Participants who interact with the robot offering creativity scaffolding (C+) generate more uncommon ideas than those who interact with the robot without creativity scaffolding (C−).

#### Post-test

We conducted an open-ended descriptive post-test interview with all participants in order to understand how they perceived the creation process.

Q1. Can you describe what you made today?
Q2. How do you think Jibo was helpful to you?
Q3. How do you think Jibo can be of more help?
Q4. Do you think Jibo had any creative ideas?

In order to provide transparency about the robot’s abilities, participants were briefed about the WoZ nature of the study and how the robot was being controlled by human instructors.

#### Data Collection and Measures

All interactions by the instructors on the desktop app were logged on to the computer along with time stamps. All tablet interactions on the WeDo application by the child were logged on to the Android tablets. We used a birds-eye view camera to record the video and audio of the interaction.

Two reviewers watched videos of the interaction and reported the creativity exhibited and the novelty of ideas. The reviewers were blind to the child’s study condition as well as the hypotheses, but were familiar with the WeDo construction activity. We used the *fluency* of ideas, *novelty* of ideas, the Unusual Uses task (Silvia, 2011), and divergent thinking as bases for measuring creativity in this task. The following three behaviors were used as metrics of creativity:

1. *Number of use cases for the rover.* We counted the number of unique applications children came up with in the free exploration time as a measure of creativity. For instance, children utilized the toolkit robot’s motion sensor and programmed an obstacle course, or used the waving of their hand to display their image. This measure is inspired by the *fluency* and originality of ideas measured (Runco and Acar, 2012), and was calculated by observing the video stream of the interaction.
2. *Number of new programming blocks used.* The instructions teach children how to use some blocks, such as the condition, motor, sensor, start and stop. Additionally, the WeDo programming interface has many different blocks that can be used in different ways, such as the image block, the sound block, other motor blocks, the text block, loops, etc. This measure is inspired by *originality* as a measure of creativity, and was calculated by analyzing the datalog of the tablet interactions.
3. *Commonality.* For each of the new use cases or application ideas of the rover, we determined how uncommon the idea was. We grouped and coded all ideas that were identical or similar, such as “obstacle course” and “lego path.” We then looked at the frequency of that idea in the data. For participants with multiple applications of the rover, we took an average of the two frequencies to report commonality. If an idea is uncommon, or deviates from the typical ideas of the group, they count as more creative (Runco and Acar, 2012). The frequencies of each application idea are inversely proportional to creativity. This measure is inspired by *divergent thinking* measures, which look at deviations from the group’s trends.

For condition analysis, we calculated numerical values for each of the three metrics by coding the video recording of each interaction. We conducted the Shapiro-Wilk test to check for normal distribution of the data collected, and then conducted an unpaired *t*-test between the study conditions for each of these measures.

### Results

**FIGURE 8:**
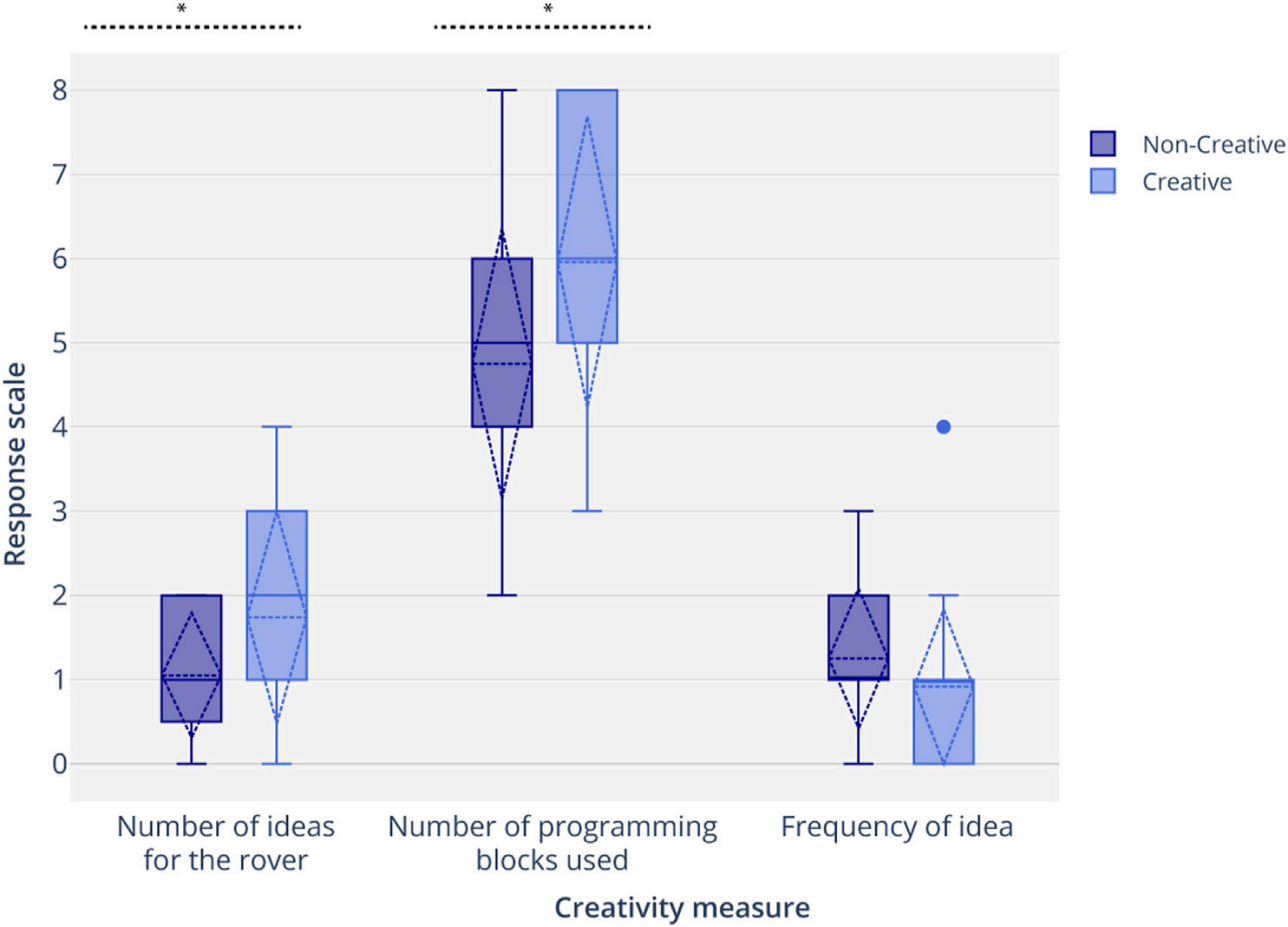
Participants in the High creative robot condition (C+) generated a significantly higher number of ideas than in the Low creative robot (C−) condition (H1). Participants in the C+ condition used a significantly higher number of programming blocks than participants in the C− condition (H2). There was no significant difference in the frequency of ideas generated in the two conditions (H3).

We tested for normality using the Kolmogorov-Smirnov test of normality on all three measures for both groups ({Number of ideas, number of new blocks, frequency of ideas}×{C+, C−}). We found that none of the groups of data differed significantly from that which is normally distributed (*p* = 0.22, *p* = 0.57, *p* = 0.55, *p* = 0.36, *p* = 0.26, *p* = 0.053). Levene’s test showed that the variances for the number of ideas were not equal, *F*(1,41) = 7.96, *p* = 0.007, and the variances for number of new blocks, and frequency of ideas were equal, *F*(1,41) = 0.312, *p* = 0.602 and F(1,41) = 0.277, *p* = 0.579. For the number of ideas, we conducted the Welch’s test assuming unequal variances, which revealed that participants expressed higher *fluency* by generating a significantly greater number of ideas for the rover (*M* = 1.42, *SD* = 1.12, *t*(36) = 1.688, *p* = 0.018). An unpaired parametric *t*-test revealed that participants in the C+ condition expressed significantly higher *originality* by using significantly more number of unique programming blocks (*M* = 5.40, *SD* = 1.80, *t*(41) = 1.682, *p = 0.013*) in the C+ condition as compared to the C− condition (Table 5; Figure 8). While participants in the C+ condition demonstrated divergent thinking and found unusual uses of the same blocks by expressing less common ideas, or more novel ideas, than the C− condition, this difference was not found to be statistically significant *(M* = 1.02, *SD* = 0.96, *t*(41) = 1.68, *p = 0.076)*. *Creativity scaffolding* offered by the robot influenced the number of ideas that children generated and the number of unique programming blocks used. Scaffolding offered by the robot led to more uncommon or atypical ideas by children, but the effect of scaffolding was not significant.

**TABLE 5:** WeDo construction task results comparing the fluency and originality of idea, and divergent thinking expressed by participants in the three study groups.

SG	Number of ideas for the rover	Number of new blocks	Frequency of ideas
High creative robot (C+) (n = 23)	1.74 ± 1.28	5.96 ± 1.77	0.82 ± 1.03
Low creative robot (C−) (n = 20)	1.05 ± 0.76	4.75 ± 1.65	1.21 ± 1.08
*Result*	*t*(36) = 1.688, *p* = 0.018	*t*(41) = 1.682, *p = 0.013*	*t*(41) = 1.68, *p = 0.076*

Participants in the High creative robot condition came up with a significantly higher number of ideas, and used a significantly higher number of programming blocks than the Low creative robot (C–) condition.

In addition to comparing participants’ creativity, we gained an insight into their perception of their creations and the robot’s role as a collaborative peer through the post-test questionnaire.

Q1. Can you describe what you made today?

We asked participants to reflect on their creations, what they used and what they learned. One participant said, “*I made him drive, make music and show an image*.” Another participant said, “*I made a LEGO robot that when you put your hands to it it’ll go back but without you touching it, and then I made a sensor and I made it make a noise*.” One participant said they made “*a spaghetti one-eyed snail cricket thing*.” These questions helped us unpack not only what their ideas were and which bricks they used, but also what their perceptions of their constructions were. Some participants also spoke about ideas they had but could not construct due to time constraints. One participant said, “*I wanted to make the rover move around and find all the walls, but there was no time*.” We also used participants’ narratives of what they made to match with the *number of ideas* metric that was reported by the blind reviewer.

Q2. Was Jibo helpful to you? How?

Eighteen participants responded with “yes, he was helpful.” Nine participants provided no reasoning. Five participants said “no” (*non-creativity scaffolding* conditions). The most common reasoning response in both study conditions was that Jibo helped them construct the rover by providing instructions. Some participants in the creative condition pointed out how, “*Jibo had cool ideas*” and “*He helped me think of other uses for the sensors*.” Multiple participants pointed out how Jibo “*told me when I was doing well, or when he liked my ideas*.” Hence, children did notice the positive reinforcement provided by the scaffolding robot. One participant in the C− condition said, “*He kind of was [helpful], but he’s super rude. Because half the time I tried to say Jibo, can you help me? He will interrupt me with something else. I tried to say good morning but he didn’t reply. it takes him a while to respond like he’s not listening*.” This highlighted some technical difficulties such as speech delays in implementing this scaffolding model. We also learned that rapport-building utterances, such as greeting utterances in the beginning, can help the children establish common ground with the robot and also help them to get acquainted with speech delays.

Q3. How do you think Jibo can be of more help?

Participants had very valuable feedback about how to make the interaction better. The most common response from both robot conditions was that it would be nicer if the robot displays the blocks to be used on his screen, and that it is difficult to understand which block he is talking about using speech alone. Unlike a human instructor, Jibo cannot point, so visuals would be very helpful. One participant in the C− condition said, “*He could have shown me other things that the rover can do*.” One participant in the C+ said, “*He could have told me what a microphone was*.” We observed that it is essential to unpack difficult terms that children might not have previously heard.

Q4. Do you think Jibo had any creative ideas?

79% of participants in the C+ condition, and 35% of participants in the C− condition, responded with “*yes*.” Hence, participants successfully perceived the expressed creativity of the robot. Some participants went on to explain why they thought that Jibo was creative. One participant said, “*Yes, he told me to make the [rover] move and can put more than one thing on the screen.*” Another participant said, “*He had cool ideas like playing music. He played fun games with me and he had great ideas and he knows that he’s smart*.” One participant also said, “*Jibo thought that I had cool ideas, and that made me happy*”, and another one said, “*Yes, he told me I can make what I want and told me my idea was great.*” Among participants that responded with “*No*” or “*Maybe*” there was typically no reasoning. One child in the C− condition said, “*Jibo knew what to do but he was not really creative*.”

## Discussion

In this work, we demonstrate how a social robotic peer’s socio-behavioral patterns can influence creativity in children in the 5–10 yr old age group. Specifically, we highlight two robot interaction patterns: *1*) *creativity demonstration*, where the robot itself demonstrates artificial creativity, and *2*) *creativity scaffolding*, where the Jibo robot supports and encourages the child’s creative thinking by asking reflective questions, providing challenges and positive reinforcements. We designed three game-based interactions that afforded different types of creativity. The robot’s interaction patterns were inspired by children’s creativity-eliciting social interactions with their peers and tutors. These interactions serve as playful ways of measuring creativity, as well as supporting children’s creative expression. In order to assess the efficacy of these interaction patterns, we conducted an accompanying investigative study for each of our game-based interactions: the Droodle Creativity game that affords children’s *verbal* creativity, the MagicDraw game that affords children’s *figural* creativity, and the WeDo construction task that affords children’s *constructional* creativity.

In the Droodle Creativity game, children emulated artificial verbal creativity exhibited by Jibo during gameplay. Participants who interacted with the high creative robot expressed more ideas, more diverse ideas, and highly creative ideas in the Droodle Creativity game, as compared to participants that interacted with the low creative robot. Similarly, in the Magic Draw Game, children emulated artificial figural creativity exhibited by Jibo in a co-drawing task, which led to their drawings being measured as more creative by the TCT-DP test for figural creativity. Participants also perceived the high creative robot as highly creative. Through these two studies, we verify our first hypothesis: that children adeptly socially emulate the creative behavior of peer-like robot playmates, and this in turn fosters children’s own creative behavior. Importantly, this is a sufficiently robust enough finding that we could replicate it for two different kinds of creative expression: verbal and figural creativity.

In the second investigation, we demonstrated how a robot offering creativity scaffolding in the form of asking reflective questions, challenging the participants, and providing positive reinforcement had a positive effect on children’s creativity. Participants engaged in an open-ended activity involving constructing and programming a rover using the WeDo construction kit. Creativity was measured by the ideas that children came up with for the rover, the number of different tools (programming bricks) they used, and how unique their ideas were. Children interacting with the robot that offered creativity scaffolding scored significantly higher on the number of different ideas and different programming bricks used. They also scored higher in the uniqueness of their ideas; however, that difference was not statistically significant. Hence, we could establish evidence that children can learn creativity from a social agent by emulating the agent’s creative behaviors and by the agent scaffolding their creative learning, which informs the design of pedagogical embodied tools to foster creativity.

The WeDo construction task utilized a predictive scaffolding model. This paves the way toward the development of fully autonomous robot scaffolding systems for tailoring personalized learning to different students and contexts. Over time and over several playtests, the model is able to reinforce itself depending on whether the instructor accepts or rejects its suggestions, eventually leading to minimal error rates. Replicating a human instructors’ scaffolding into an artificial agent can be beneficial for personalized assistance when the teacher is not present or when there are many students per teacher. This scaffolding paradigm could be used in the context of other activities.

It is important to be wary of the shortcomings of such a suggestion based system for instructors. While these recommendations make it easy for instructors to provide help and scaffold children’s creative learning, they also inhibit the instructor’s original thought and manner of scaffolding, which holds high value. A more autonomous model would be built from data collected from multiple instructors with a diverse set of backgrounds and expertise, all of whom could instruct several students from a diverse set of backgrounds. Further, the model should be able to adapt based on an instructor’s usage while allowing enough space for original thought. While the current interface does allow instructors to reject the model’s recommendation and instead generate new Jibo utterances, it can still influence the instructors’ decision making process, and cause them to conform to commonly used instructions, which may be counterintuitive to promoting creativity. In our work we suggest two interaction patterns of social robots that effectively foster creativity in young children: creativity demonstration and creativity scaffolding using questions, questions and ideations. We position social robots as CSTs in collaborative activities with children that can leverage the benefits of having a co-located peer to create and socially interact with, and a digital CST that adapts to the person’s creation style. We also add to the literature of HRI suggesting that children emulate a robot’s learning behavior and extend it to verbal, figural and constructional creativity. Through our observations from playtests, iterative game design, study results and post-test questionnaires, we formulated evidence-based design guidelines as well as additional recommendations from researchers’ reflections for designing creativity supporting social agents that we have outlined in the next section. These recommendations could benefit pedagogical researchers, educators and HRI and HCI practitioners designing social agents for stimulating creativity, especially in children.

### Design Recommendations

#### Evidence-based Design Guidelines

In this section, we suggest the following interaction design recommendations for social agents to support children’s creativity based on our empirical findings:

1. The social agents should demonstrate the creativity behaviors that the designers aim to foster. We observed that children can learn verbal and figural creativity by emulating a social robot’s creative behaviors across all three tasks. Hence, while designing social robots as pedagogical tools, we must ensure that they express the desired creative behavior that researchers aim to foster in children. The expression of creativity is context-dependent (such as generating creative drawings) and can be supported by social behaviors such as reflecting on the artifact generated, or making the creative process transparent through dialogue.
2. Make use of reflective questioning and challenges. Asking reflective questions about the children’s actions aids their metacognition and creative thinking. Through the scaffolding study, we learned that instructors who were controlling the robot remotely chose to use many reflective questions as robot speech prompts to provide creativity scaffolding for children. Providing children with optimal levels of difficulty encouraged them to solve problems creatively. In the WeDo construction activity, the scaffolding robot provided challenges to the child, such as, “Can you think of other uses of the same sensor?” or “Do you think that’s the best way to do it?” These questions were followed by students exploring creative uses of the objects beyond the first use that they imagined which encouraged flexibility. For instance, one child utilized the motion sensor’s ability to detect motion and turn to create an obstacle path for her rover. We observed that children who interacted with the creativity scaffolding robot, which asked reflective questions, exhibited higher levels of creativity in the task.
3. The agent should generate unique and frequent ideas during the interaction. In addition to asking reflective questions and posing challenges, the robot could also demonstrate new idea generation. During the WeDo task, when the robot suggested, “You can use the picture icon to display images when the robot senses an object,” the child subsequently used the feature to display the preset images. The child then uploaded their own image and made the rover move towards them. When the rover detected the child as the obstacle, it displayed the child’s photograph. Children not only incorporated the robot’s ideas, but also built upon them. In the Droodle Creativity game, we observed that the robot’s idea generation behaviors were emulated by the child, both in terms of the fluency and originality of ideas generated.
4. Provide positive reinforcement to children when they create. Children in all three tasks commented how the robot said “Good job!” or other similar positive comments after they completed the task. Positive reinforcement after creative behaviors has a strong influence on children. Children often form relationships with social robots, which lead to increased learning gains (Westlund et al., 2018) and getting positive validation upon exhibition of creativity encourages them to be more creative.

#### Additional Recommendations From Researcher’s Reflections

We incorporated several design principles from background research in CSTs and iteratively designing our child-robot interactions. While we found these design decisions to be beneficial for children’s creativity and had a positive combined effect, we did not analyze the effectiveness of each of these recommendations, and future work is required to disambiguate individual effectiveness. We present these as additional design recommendations for designing creativity scaffolding interventions for children:

1. Co-design interactions with instructors. Co-designing scaffolding robot interactions with instructors helped us personalize interactions for the students, and incorporate instructors’ teaching experience into the robot’s behaviors. Instructors were particularly helpful in designing the interaction GUI; while the pre-set GUI speech prompts were grounded in historic scaffolding commands used by other instructors, we found that teachers often needed to personalize interactions during unique occurrences. Having the option of typing new commands in the GUI text-box afforded instructors that flexibility. This hybrid interface equipped teachers with historically useful commands, reducing their cognitive load during the task, and gave them the ability to create their own.
2. Scaffolding must be grounded in tasks and materials. In the creativity scaffolding task, we started with providing teachers with generic scaffolding prompts such as “What else can you make?” or “What is another way to do that?” However, teachers provided us with feedback that the scaffolding prompts needed to be specific to the task (construction) and materials (blocks). For instance, they used the prompt, “How else can you use that sensor to make the rover move?” This grounding in the collaborative task portrayed the robot as a context-aware scaffolding agent. Hence, while designing scaffolding interactions such as challenges or reflective questions, we found it beneficial to ground the interactions in the task’s context instead of generic interactions. However, this reduces the scalability of these interactions across tasks.
3. Agents must scaffold, but not impose. Scaffolding through social interactions can be powerful but has the potential to inhibit creativity. Interactions such as idea generation in collaborative tasks must be designed such that the agent does not impose their ideas on the child, nor intrude upon the children’s creative space. For instance, in the WeDo scaffolding tasks, we observed that teachers who controlled the robot suggested ideas related to the child’s working idea, and while the child worked on an idea, they did not interfere. This delicate scaffolding can be a challenge to execute in fully autonomous interactions. Providing scaffolding only when the child asks, or when the child is stuck, could be a beneficial approach. Further, care should be taken to not interrupt children’s creative process; in the MagicDraw interaction, where players had a fixed time to draw for each turn, one participant reported displeasure for the robot interrupting their drawing.
4. Design game-based interactions with peer-like social agents. Designing game-based child-robot interactions enabled us to position the robot as a collaborative playful peer. This made the interaction fun for children as reported in the post-test, and children were engaged throughout the games. We designed game tasks with no fail state in order to provide an outlet for unconstrained creative thinking and encourage divergent thinking. Since assessment is shown to hinder creativity, we refrained from providing any assessment during the interaction. Game-like interactions made the tasks engaging for young children and allowed for a safe space for failure.
5. Center task around creation of artifacts. In order to maximize space for creative thinking, we designed the tasks around the creation of artifacts rather than the completion of a specific deliverable. In accordance with literature showing evaluation’s negative effect on creativity, these artifacts were designed to have no set of “correct” answers, supporting creative exploration without an end goal. To ground robotic scaffolding in the context of the task, we provided a limited set of materials that can be used creatively to produce an unlimited number of artifacts. For instance, in the WeDo construction task, one student wanted to create two rovers but was limited to one controller. They wired the sensor of the second rover from the first rover’s controller and called it a “parasite” rover.
6. Leverage collaboration as an agent behavior and game mechanic. Since collaboration has a positive influence on creativity, we must ensure that the child-agent interactions are collaborative in nature and the robot acts as a collaborative peer instead of a competitive one, which hinders creativity in children. The collaborative nature of the interaction was made explicit in robot speech, such as, “Today we will program a robot together.” Within our tasks, we framed the social robot agent as a peer helping the child do their best creative work, and the majority of children perceived Jibo as a collaborator rather than a competitor. Collaboration is among the most prominent social factors that positively influence creativity. Careful consideration must be given to interactions with the agent in particular, in order to ensure that children see it as a partner rather than as a competitor, which can hinder their creativity. Introduction of the robot and the task can be leveraged to position the robot as a collaborative peer.

## Conclusion

In this work, we posit social robots as CSTs for children in collaborative tasks. We studied the effects of an autonomous social robot’s verbal and nonverbal interactions on children’s creativity as measured by three collaborative game-based child-robot interactions. We observed that both creativity demonstration and creativity scaffolding offered by the social robot had a positive effect on children’s creativity in verbal, figural and constructional creativity tasks. This work contributes to the design of game-based child-robot interactions that afford creativity, provides evidence for the efficacy of these interactions and provides guidelines for designing social embodied agents to foster creativity in young children. These findings are valuable to game designers creating game-based interactions to foster children’s creativity, as well as HRI and HCI practitioners leveraging social agents as CSTs.

Since robots are already being used in classroom settings as learning peers and personalized tutors, it is imperative to think about how their behaviors can influence children’s learning behaviors, such as creative thinking. While social robots are not the only way to provide creativity support through behavioral modeling, they certainly are a compelling way given their social nature. Effort must go into designing the agents’ behavior such that they exhibit creativity and scaffold the child’s creativity as a peer or a tutor. Embodied AI agents have the potential to use generative modeling techniques to express different forms of creativity through generating media such as drawing, poetry, art styles, patterns, physical body movements, etc. They are also socially emotive and can express the social interactions that accompany creativity such as reflection, inquisitiveness and positive affect. This work opens up opportunities to explore how these different forms of artificial creativity can be embedded into tools that children use and interact with, and help them be more creative.

Recent works have demonstrated how robots can help creativity, as a co-present partner and through social interactions. In this work, we suggest two interaction patterns of social robots that we observed to effectively foster creativity in young children: creativity demonstration and creativity scaffolding using questions, questions and ideations. We add to the literature of HRI suggesting that children emulate robots’ learning behaviors, and that this phenomenon extends to creativity. We also contribute to the field of Creativity Support Tools by positioning social peer-like robots as a creativity support peer in collaborative activities. This contribution is not only valuable for HRI practitioners, but also other interactive AI agents, such as conversational agents. Creativity supporting social robots combine the benefits of having a co-located peer to collaboratively create with and socially interact with, and a digital CST that adapts to the user’s creation style. Leveraging generative AI models now allow for expressing creativity in several modalities, which robots can successively leverage. Further, we elaborate the design of a scaffolding mechanism by learning from human scaffolders through one construction activity. This approach can be generalized to other activities.

While introducing an extrinsic factor in the form of a social robot, we must ensure that it does not come across as an evaluator; classroom research has demonstrated how extrinsic factors such as evaluation, competition and unrealistic expectations can potentially inhibit creativity, instead of fostering it (Torrance, 1967). In the scaffolding GUI design, we provide instructors with a predictive interface that helps them scaffold the child for creative learning; however, this suggestion model also limits creativity and personalization of teaching style from the instructors. To tackle this issue, we must also aim to build personalized scaffolding models that take input from every teacher and personalize over time in both the content and style of learning.

In our work, we chose a wide age range (5–10 yr) and we did not analyze differences across age ranges. This is a limitation of the current work, and future analysis needs to be run with narrower age ranges to determine the efficacy of the intervention on different age groups. Another limitation of this work is that these robot interactions lead to an increase in creativity within the narrow constructs of these tasks, and may not scale to every creative task, or to students’ life outside of these tasks. There are also countless ways of expressing creativity such as poetry, storytelling, painting, music, etc., that are not explored in these tasks. This work also defines a limited scope of creativity in terms of fluency, novelty and value of ideation. Creativity encompasses a much wider array of behaviors (such as divergent thinking) that can be explored using other interactions. Furthermore, while all these interactions currently focus on one-on-one child-robot interaction, we must strive towards designing interactions that involve multiple children because collaboration with peers forms a major part of creative learning.

Finally, while this work evaluates the role of the robot’s creativity fostering behaviors, it does not evaluate the benefits of embodiment over other non-embodied agents such as computers or voice agents. Previous research has found that adults did not show significant gains in creativity merely in the presence of a social robot (Alves-Oliveira et al., 2019). In future work, we aim to study the combined effect of embodiment and creativity scaffolding behaviors by running a 2 × 2 study ({embodied, non-embodied}×{scaffolding, non-scaffolding}) (Devasia et al., 2020). In order to evaluate whether robots are really that social, future work is required to assess the creativity effects of human peers vs robots.

While this work makes use of Jibo as the social agent, these interactions are learning tools that can foster creativity in classrooms and homes independent of Jibo. These games also serve as game-based creativity assessment measures.

We designed a creativity scaffolding paradigm for the WeDo construction task. This model currently supports a semi-autonomous scaffolding system, where a human controls the robot using a remote control desktop program. In the current version of the robot control interface, which lets instructors control the robot remotely, we can incorporate ASR to use the instructors’ speech to control the robot’s speech. Moreover, collecting more data about how instructors use the program can help us build a fully autonomous model of scaffolding. While this approach can be used to design interactions for an autonomous or semi-autonomous system, the timing of the interactions and during-study improvisation are reliant on children’s actions in the task and interactions with the robot. Current status of natural language understanding and computer vision limit a complex understanding on the scene, and hence a similar fidelity of robotic interactions are challenging to currently implement in a fully autonomous system. However, as we demonstrated, a semi autonomous system (where the system suggests interactions and the human decides the timing of the prompts) is feasible.

Another limitation of this work is that all activities are self-contained and involve single interactions. In future work, it would be valuable to evaluate creativity transfer from one activity to another, and even in the absence of the robot in the long-term. Design recommendations from this work can be incorporated in several creativity support tools such as computer games, voice agents, tablet apps, embodied tools, space design, etc. Finally, advances in generative modeling techniques enable us to create child-robot interactions supporting multiple modalities of autonomous creative expression.

## Data Availability

The raw data supporting the conclusions of this article will be made available by the authors, without undue reservation.
